# Supplementary material for: Effectiveness of A Nurse-Led Multimodal Intervention in Preventing Blood Culture Contamination: A Before-and-After Study
Source: Healthcare (Basel). 2024 Aug 31;12(17):1735. doi: 10.3390/healthcare12171735 (PMC11395113; doi:10.3390/healthcare12171735)
Supplement: Supplementary file 1 [file healthcare-12-01735-s001.zip › healthcare-3161736-supplementary.pdf]

Hospital Distrital da Figueira da Foz, E.P.E.

UNIDADE LOCAL DO PROGRAMA DE PREVENÇÃO E CONTROLO DE INFEÇÃO E DE RESISTÊNCIA AOS ANTIMICROBIANOS

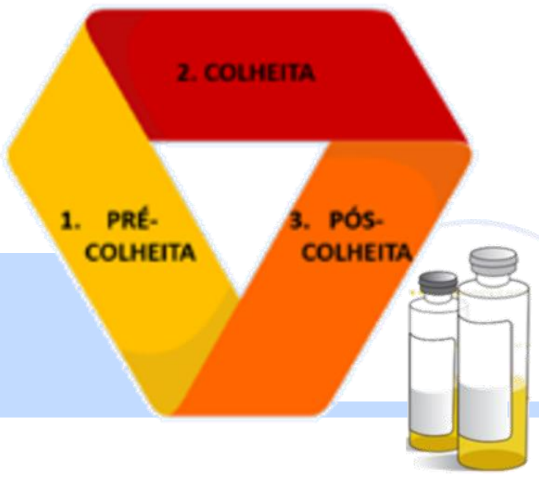

HEMOCULTURA ETAPAS DA COLHEITA

ACEDE AQUI AO VÍDEO DO PROCEDIMENTO

1. PRÉ-COLHEITA

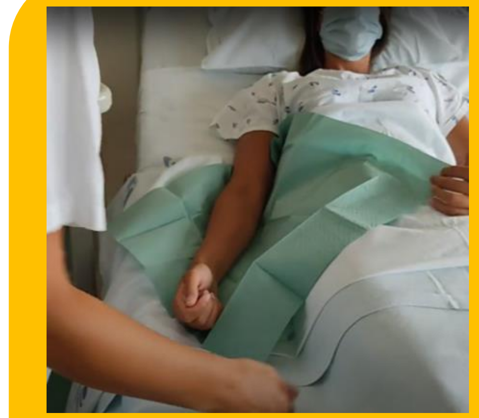

INFORMAR O UTENTE SOBRE O PROCEDIMENTO OBTER CONSENTIMENTO

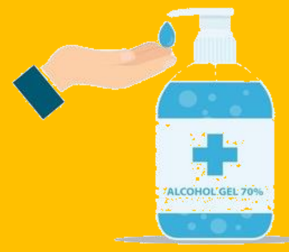

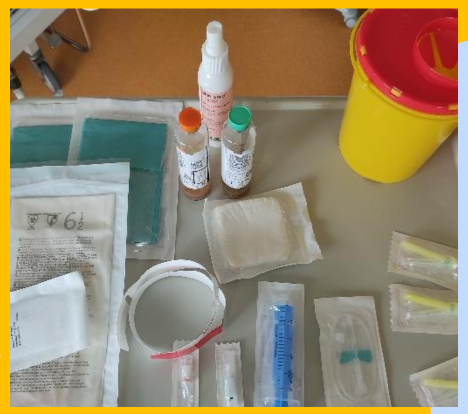

REUNIR O MATERIAL NECESSÁRIO

- 1 *set* de garrafas – 1 aeróbio e 1 anaeróbio
- Antisséptico – clorohexidina de base alcoólica a 2%
- Compressas estéreis, luvas estéreis, garrote de uso único
- 3 agulhas de colheita (ou 1 *butterfly* com adaptador e duas agulhas)
- 1 adaptador de membrana e 1 seringa de 20 ml
- 1 campo estéril e 1 campo limpo impermeável
- 1 penso com compressa
- Contentor imperfurável (acondicionamento de corto-perfurantes, grupo IV)
- Contentor para o acondicionamento de resíduos do grupo I e II e do grupo III

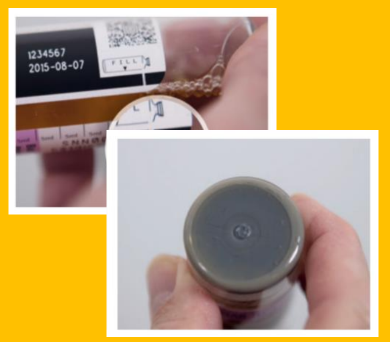

VERIFICA O PRAZO DE VALIDADE E O FUNDO DAS GARRAFAS (DEVE SER CINZENTO)

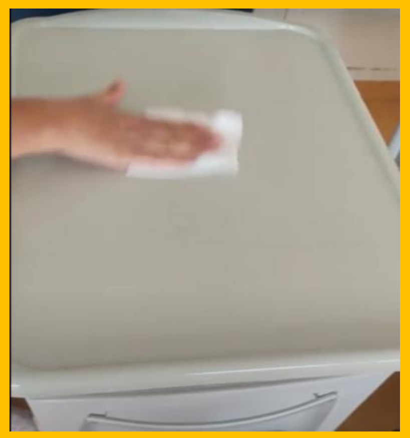

DESINFETAR A BASE DE TRABALHO

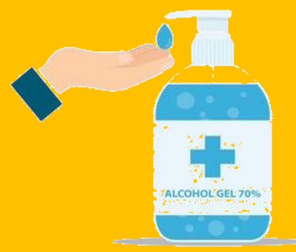

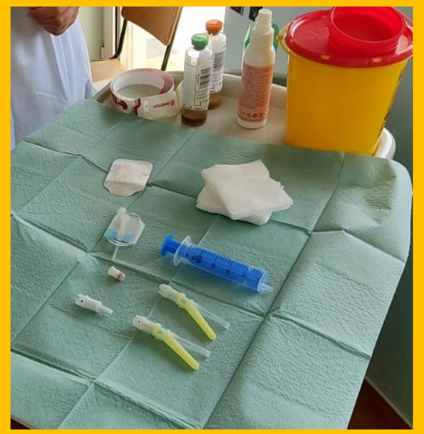

PREPARAR O MATERIAL NUM CAMPO ESTÉRIL

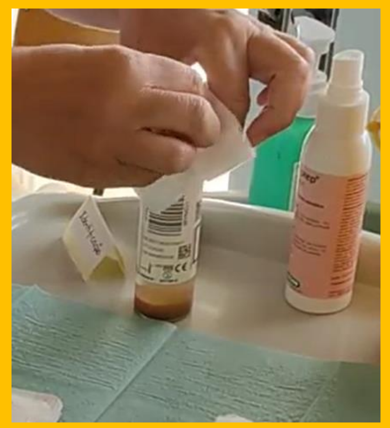

DESINFETAR A BORRACHA DAS GARRAFAS (ÁLCOOL A 70º OU CLOROHEXIDINA DE BASE ALCOÓLICA 2%)

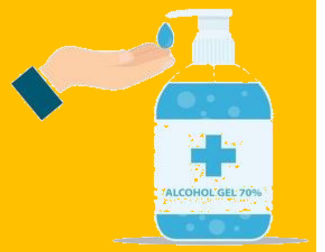

2. COLHEITA

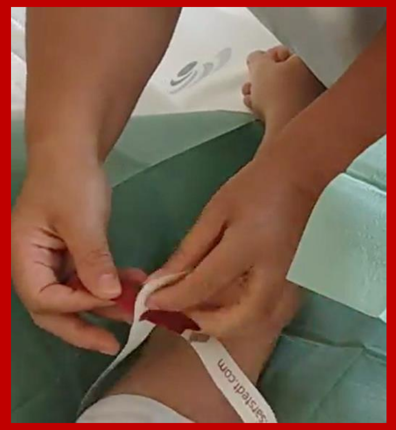

COLOCAR CAMPO LIMPO IMPERMEÁVEL POR BAIXO DO BRAÇO COLOCAR GARROTE

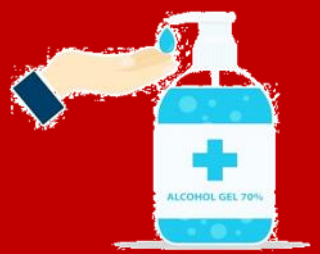

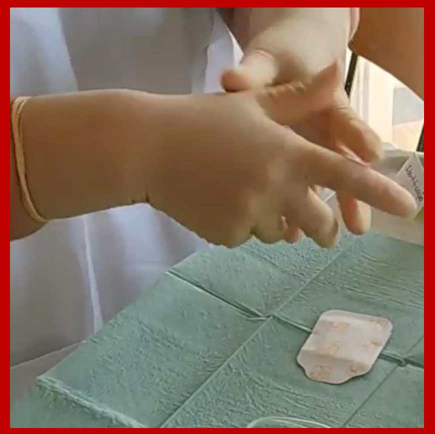

CALÇAR LUVAS ESTÉREIS

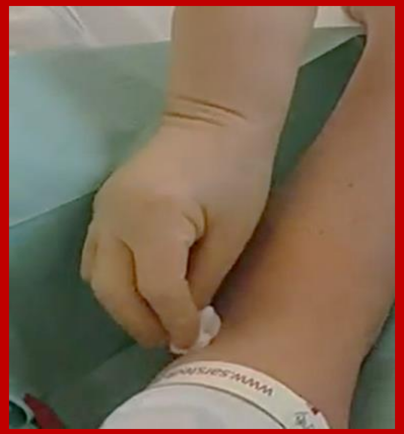

ANTISSÉPSIA DA PELE (CLOROHEXIDINA DE BASE ALCOÓLICA A 2%) DEIXAR SECAR

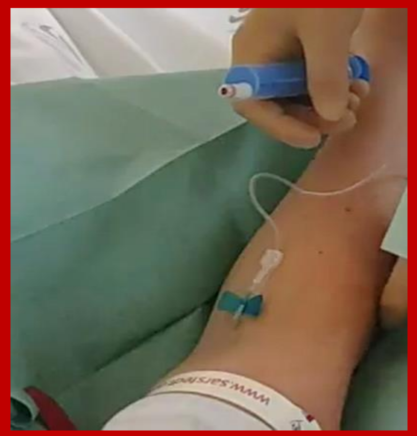

PROCEDER À FLEBOTOMIA, ADAPTAR A SERINGA E ASPIRAR

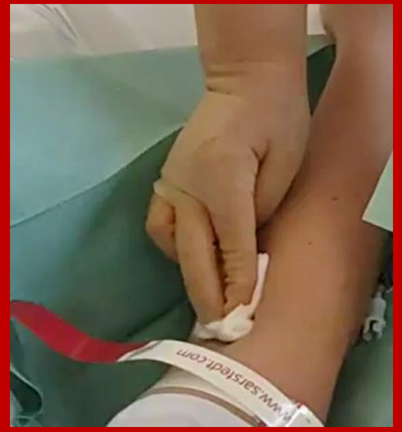

DESGARROTAR E RETIRAR A AGULHA. APLICAR PRESSÃO

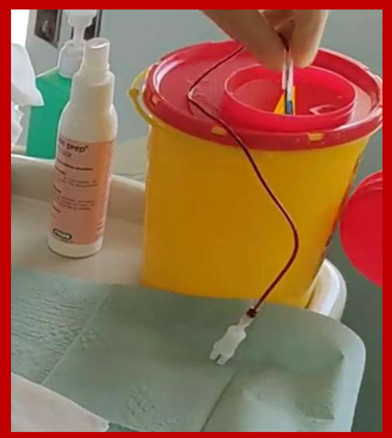

ACONDICIONAR AGULHA EM CONTENTOR IMPERFURÁVEL (GRUPO IV)

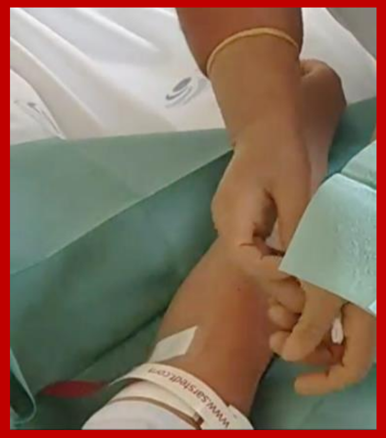

APLICAR PENSO

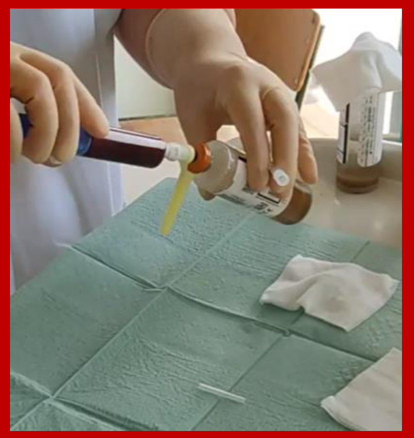

INOCULAR FRASCOS ANAERÓBIO E AERÓBIO (USANDO UMA AGULHA NOVA PARA CADA FRASCO)

3. PÓS-COLHEITA

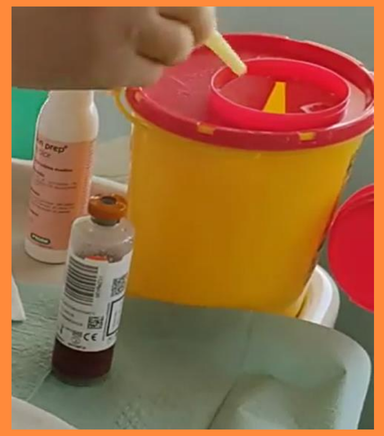

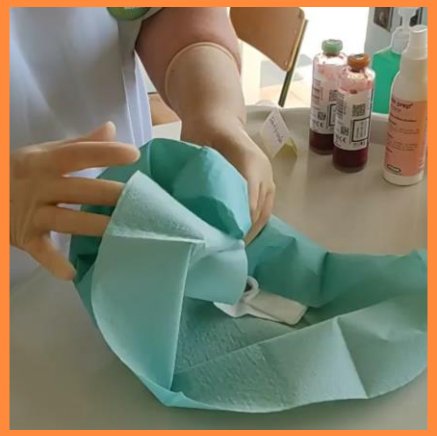

ACONDICIONAR TODOS OS RESÍDUOS DE ACORDO COM A SUA CLASSIFICAÇÃO:
GRUPO IV (CONTENTOR IMPERFURÁVEL);
GRUPO III (SACO BRANCO)

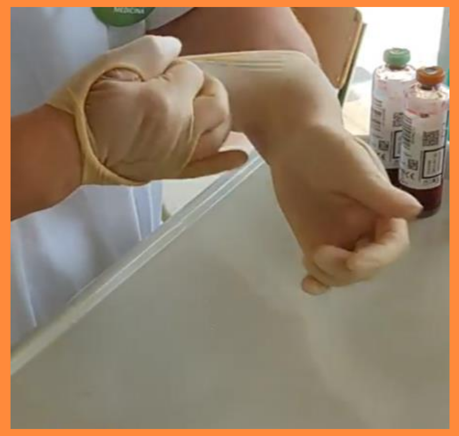

RETIRAR LUVAS E ACONDICIONAR COMO RESÍDUO GRUPO III

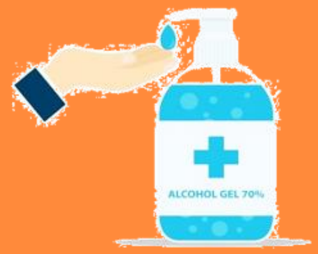

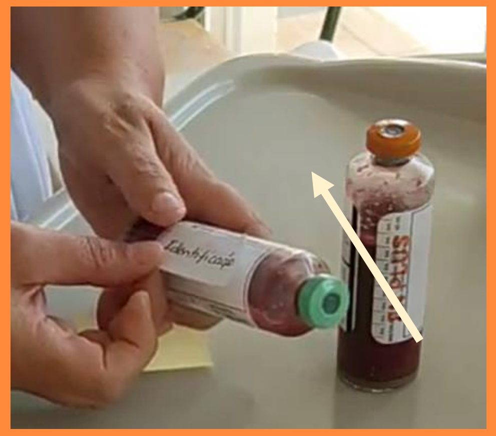

APLICAR ETIQUETA IDENTIFICATIVA, SEM TAPAR O CÓDIGO DE BARRAS

RESPEITAR O NÍVEL DA GARRAFA

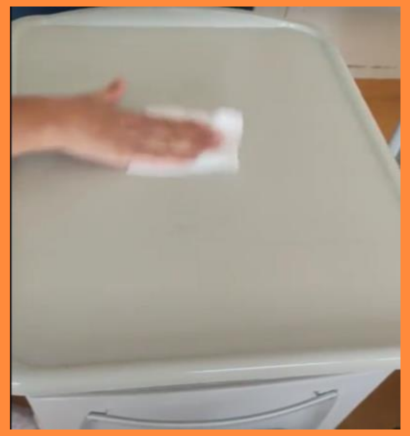

DESINFETAR A BASE DE TRABALHO

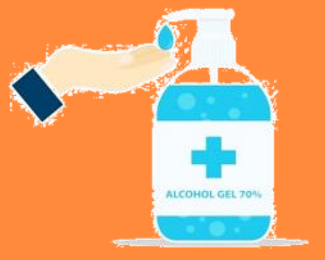

APÓS DEIXAR A UNIDADE DO DOENTE, ENCAMINHAR AS AMOSTRAS PARA O LABORATÓRIO.

Vol. de sangue:
- adulto, 10ml em cada garrafa
- criança, de acordo com o peso

Se houver outras colheitas, a hemocultura é sempre 1º

ACEDE AQUI À APRESENTAÇÃO COM DESCRIÇÃO DE TODAS AS ETAPAS DE COLHEITA
